# Morin Alleviates Fructose-Driven Disturbance of Podocyte Mitochondrial Energy Metabolism by Inhibiting Adenosine 5′-Monophosphate Deaminase Activity to Improve Glomerular Injury

**DOI:** 10.3390/ph18121883

**Published:** 2025-12-12

**Authors:** Yingzhi Yang, Ziyan Wan, Luyi Huang, Ziang Zhou, Wanru Wang, Yu Xing, Shijie Li, Yufan Du, Jiufang Huang, Yanqing Wu, Mengyu Fan, Jiahuang Li, Lingdong Kong, Dongmei Zhang

**Affiliations:** 1 State Key Laboratory of Pharmaceutical Biotechnology, School of Life Sciences, Nanjing University, Nanjing 210023, China; yangyingzhi@smail.nju.edu.cn (Y.Y.); wanziyan@smail.nju.edu.cn (Z.W.); huangluyi@smail.nju.edu.cn (L.H.); zhouziang@smail.nju.edu.cn (Z.Z.); wangwanru@smail.nju.edu.cn (W.W.); xingyu@smail.nju.edu.cn (Y.X.); 181850073@smail.nju.edu.cn (S.L.); 181850032@smail.nju.edu.cn (Y.D.); jiufanghuang@smail.nju.edu.cn (J.H.); 522025300045@smail.nju.edu.cn (Y.W.); mengyufan@smail.nju.edu.cn (M.F.); 2 School of Biopharmacy, China Pharmaceutical University, Nanjing 211198, China; lijiah@cpu.edu.cn

**Keywords:** morin, fructose, podocyte injury, adenosine 5′-monophosphate deaminase, purine nucleotide cycle, mitochondrial energy metabolism

## Abstract

**Background/Objectives:** High fructose consumption is a significant risk factor for glomerular podocyte injury. This study aimed to identify the underlying mechanism of fructose-induced podocyte injury and explore the protective effect of the natural polyphenol morin. **Methods**: In vivo, high-fructose-diet-fed rats were used to evaluate podocyte injury through ultrastructural structure analysis, urinary albumin-to-creatinine ratio (UACR), and synaptopodin expression. In vitro, adenosine 5′-monophosphate deaminase (AMPD) expression and activity, mitochondrial function, and glycolytic flux were measured in mouse podocyte clone-5 (MPC5) exposed to 5 mM fructose, with molecular docking and siRNA interference assays validating morin’s regulatory role. **Results**: High fructose significantly increased AMPD activity in the purine nucleotide cycle (PNC), leading to mitochondrial dysfunction and a compensatory activation of glycolysis in podocytes. Morin effectively mitigated podocyte injury and suppressed the upregulation of AMPD activity, potentially through targeting AMPD2, as evidenced by molecular docking, which demonstrated a strong binding affinity between morin and AMPD2. Similarly, AMPD2 knockdown markedly alleviated mitochondrial impairment and glycolysis activation, confirming the pivotal role of AMPD2 in fructose-induced podocyte injury. In high-fructose-diet-fed rats, morin substantially improved ultrastructural damage, as shown by reduced podocyte foot process effacement, decreased UACR, restored glomerular synaptopodin expression, and suppressed AMPD activity in the renal cortex. **Conclusions**: Morin alleviated high-fructose-induced podocyte injury by inhibiting AMPD activity in the PNC, highlighting AMPD2 as a potential therapeutic target for podocyte injury caused by high fructose intake. This study provides novel mechanistic insights into how morin counteracts mitochondrial energy disturbance in podocyte injury.

## 1. Introduction

Glomerular podocyte injury exacerbates the progression of kidney disease to end-stage renal failure. Clinical and experimental evidence suggests that high-fructose diets cause metabolic syndrome. In our previous study, the ultrastructure of the mitochondria was severely disrupted in glomerular podocytes from rats fed with high fructose [1]. Meanwhile, the basal oxygen consumption rate (OCR), ATP generation, and maximal respiration were significantly reduced in human podocytes cultured with high fructose [1]. The mechanisms that may trigger mitochondrial dysfunction are worth exploring.

Distinctly from the glucose metabolic pathway, fructose is rapidly converted to fructose-1-phosphate by fructokinase due to a deficiency of negative feedback, leading to depletion of intracellular ATP [2]. Podocytes, the outermost layer of the glomerular filtration barrier, demand massive energy to maintain the intricate morphology of interdigitating foot processes and functions [3]. Actin disarrangement and cell death have been induced in cultured mouse podocytes by reducing ATP content with 2-deoxyglucose (2-DG) and antimycin [4]. In addition, decreased cellular ATP content has been detected in angiotensin II-treated human podocytes [5], and inhibition of glycolysis by knockdown of pyruvate kinase M2 further aggravates ATP deficiency and exacerbates podocyte injury [6]. Therefore, the disturbance of energy metabolism in podocyte injury remains to be elucidated under high fructose exposure.

The purine nucleotide cycle (PNC) is widely recognized for its role in maintaining energy homeostasis in skeletal muscle [7,8,9] and governing de novo purine synthesis in all tissues [10]. The PNC is a combination of three reactions catalyzed by adenosine 5′-monophosphate deaminase (AMPD), adenylosuccinate synthetase (ADSS), and adenylosuccinate lyase (ADSL), and functions as a pathway for the reversible deamination of AMP [11]. the PNC is activated as a compensatory approach to replenish consumed ATP and maintain the ratios of [ATP]: [AMP] and [ATP]: [ADP] in rat skeletal muscle [7,9]. Furthermore, knockdown of *Adss* or *Adsl* in mouse *Famin*^p.254I^ M0 macrophages reduces the OCR and extracellular acidification rate (ECAR) [12], which reflects decreased oxidative phosphorylation and glycolytic activity, respectively. In AMPD2-deficient mice, hepatic gluconeogenesis is reduced, and the expressions of several genes involved in fatty acid and cholesterol metabolism are dysregulated [13]. In the kidney, the PNC has been primarily characterized as a regulator of ammonia generation and acid–base homeostasis [14], with its activation observed under pathological stressors, including acidosis and hypoxia [15,16,17]. However, its specific role in renal energy metabolism remains unexplored. Given the established connection between PNC function and cellular energy dynamics, we suppose that the PNC may function as a key mechanistic link in fructose-induced podocyte injury.

Morin (3,5,7,2′,4′-pentahydroxyflavonoid), a natural flavonol abundant in various fruits and vegetables, possesses multiple pharmacological activities, including antioxidant, anti-inflammatory, and anti-cancer [18]. Morin is reported to promote the activity of mitochondrial complexes I, III, and V in HT22 cells treated with iodoacetic acid [19]. Furthermore, in male Wistar rats with isoproterenol-induced myocardial infarction, morin restores the activities of mitochondrial TCA enzymes. Our previous study demonstrated that morin significantly ameliorated kidney injury and improved renal inflammation, insulin resistance, and kidney injury in rats [20]. Whether morin influences the PNC to redress the imbalance in energy metabolism and mitigate podocyte injury remains uncharted.

In this study, an endeavor was devoted to exploring the role that the PNC performs in high-fructose-induced podocyte injury and the mechanism by which morin improves an energy disturbance in the podocytes. Evaluation of the activities of three enzymes in the PNC combined with siRNA interference in vitro identified AMPD, especially AMPD2, as the principal mediator of high-fructose-induced podocyte injury. Through integrating in vivo and in vitro models, we demonstrated the protective effects of morin against fructose-induced podocyte injury through regulation of AMPD activity, with molecular docking indicating a potential direct interaction between morin and AMPD2. These findings provide new insights into therapeutic strategies for metabolic-syndrome-associated glomerular dysfunction.

## 2. Results

### 2.1. Morin Ameliorates High-Fructose-Induced Podocyte Injury and Mitochondrial Dysfunction in Mouse Podocyte Clone-5 (MPC5)

**Figure 1:**
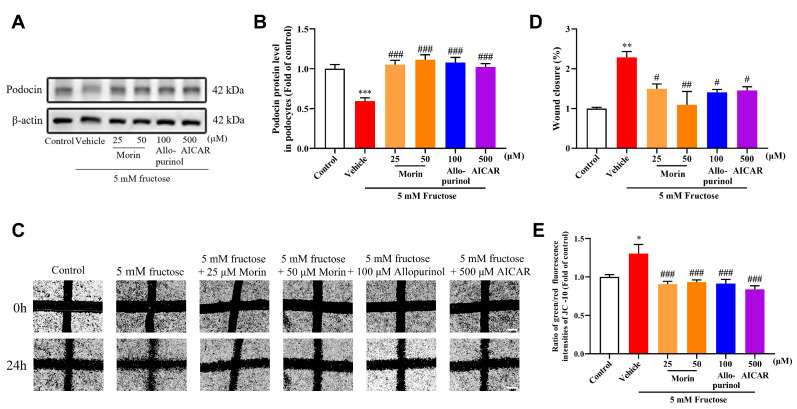
Effects of morin on fructose-induced podocyte injury and mitochondrial dysfunction in MPC5. (**A**,**B**) Protein expression level of podocin was determined by Western blot and quantified by Image J. β-actin was chosen as loading control (n = 8). (**C**,**D**) Podocyte motility was detected by wound-healing assay. Scale bar = 500 μm (n = 3). The data conformed to normal distribution; one-way ANOVA with Dunnett’s multiple comparison test was used to assess statistical differences. (**E**) Mitochondrial membrane potential was detected by JC-10 assay (n = 5–6). * *p* < 0.05, ** *p* < 0.01, *** *p* < 0.001 compared with normal control group, ^#^ *p* < 0.05, ^##^ *p* < 0.01, ^###^ *p* < 0.001 compared with fructose group.

Morin significantly reversed the downregulation of podocin in podocytes induced by 5 mM fructose for 72 h (Figure 1A,B). Meanwhile, the abnormal motility of the podocytes elicited by high fructose was also attenuated by morin (Figure 1C,D). Furthermore, the mitochondrial membrane potential, as assessed by the JC-10 assay, increased significantly following treatment with high fructose, but this effect was mitigated by morin (Figure 1E). These results indicate that morin could restore high-fructose-induced podocyte injury and mitochondrial dysfunction.

### 2.2. Morin Inhibits the Fructose-Induced Enhancement of AMPD Activity in MPC5

**Figure 2:**
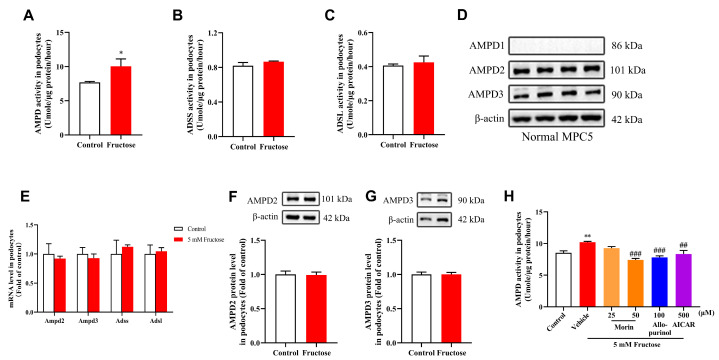
Effects of morin and AICAR against fructose-induced enhancement of AMPD activity in MPC5. (**A**) Phenol/hypochlorite reaction was used to determine AMPD activity in high fructose-stimulated podocytes (n = 5–6). (**B**,**C**) The enzyme activities of ADSS and ADSL in podocytes were assayed by colorimetric methods (n = 4–6). (**D**) Western blot analysis of the protein levels of AMPD1, AMPD2, and AMPD3 in podocytes (n = 6). (**E**) The Ampd2, Ampd3, Adss, and Adsl mRNA levels in high-fructose-exposed podocytes were measured by qRT-PCR (n = 3). (**F**,**G**) Western blot analysis of the protein levels of AMPD2 and AMPD3 in high-fructose-induced podocytes (n = 6). (**H**) Activity analysis of AMPD in podocytes treated with or without 5 mM fructose, 25 μΜ and 50 μM Morin, 100 μM Allopurinol, and 500 μM AICAR (n = 5–6). * *p* < 0.05, ** *p* < 0.01 compared with normal control group, ^##^ *p* < 0.01, ^###^ *p* < 0.001 compared with fructose group.

Colorimetric methods were utilized to detect changes in the activities of three enzymes in the PNC. The results indicated that fructose significantly enhanced the activity of AMPD (Figure 2A), while it had no significant effect on the activities of ADSS (Figure 2B) and ADSL (Figure 2C). There are three members in the AMPD family, namely, AMPD1, AMPD2, and AMPD3. The kidneys primarily express AMPD2 and AMPD3, according to the data obtained from The Human Protein Atlas database. Consistently, only AMPD2 and AMPD3 were detected in MPC5 by Western blot analysis (Figure 2D). qRT-PCR analysis indicated that the expression levels of AMPD2, AMPD3, ADSS, and ADSL in the podocytes did not change significantly following the high fructose treatment (Figure 2E). Meanwhile, the protein expression levels of AMPD2 and AMPD3 also remained unchanged (Figure 2F,G). These initial findings suggested that high fructose enhanced the activity of AMPD rather than changing its expression levels. More importantly, morin significantly inhibited the enhanced activity of AMPD induced by high fructose compared to the fructose group. A similar inhibitory effect on AMPD activity was also observed in the group treated with AICAR, a reported PNC inhibitor, in fructose-exposed podocytes (Figure 2H).

**Figure 3:**
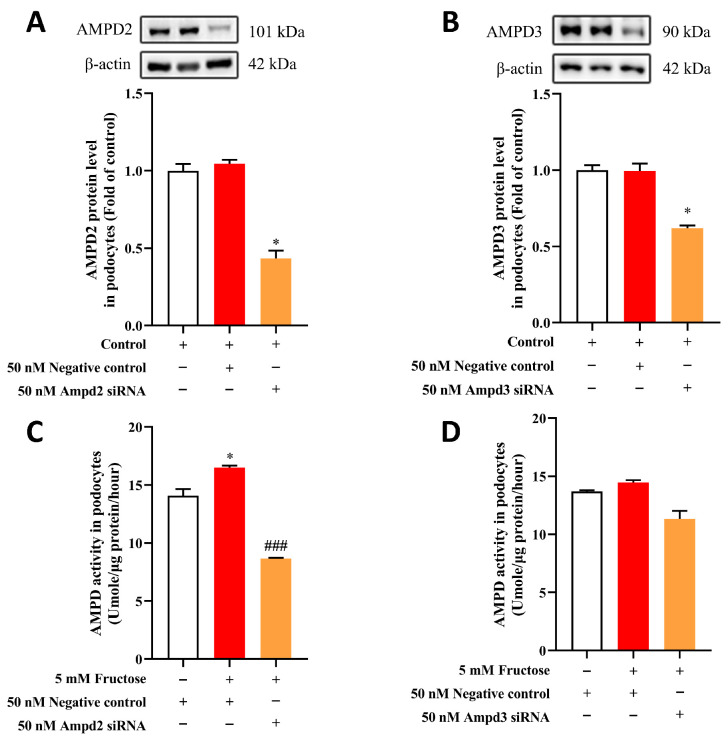
AMPD2 plays a more important role in regulating AMPD activity compared to AMPD3 in MPC5. (**A**,**B**) The protein levels of AMPD2 and AMPD3 were detected in podocytes transfected with Ampd2 siRNA, Ampd3 siRNA, and negative control siRNA (n = 4). (**C**,**D**) AMPD activity was analyzed in podocytes transfected with Ampd2 siRNA, Ampd3 siRNA, as well as negative control siRNA, and then incubated with fructose or a vehicle (n = 3). * *p* < 0.05 compared with the normal control group (transfection with negative control siRNA), ^###^ *p* < 0.001 compared with the fructose group (transfection with negative control siRNA).

The subsequent investigation aimed to determine the roles of AMPD2 and AMPD3 in podocyte injury induced by high fructose. Therefore, AMPD2 and AMPD3 siRNA were employed to interfere with the expression of AMPD2 and AMPD3, respectively. As shown in Figure 3A,B, the protein expression levels of AMPD2 and AMPD3 in podocytes noticeably decreased after transfection with the corresponding siRNA. Furthermore, the activity of AMPD in the podocytes declined more significantly in the context of silenced AMPD2 compared with AMPD3 (Figure 3C,D), suggesting that AMPD2 may play a more important role in regulating AMPD activity.

**Figure 4:**
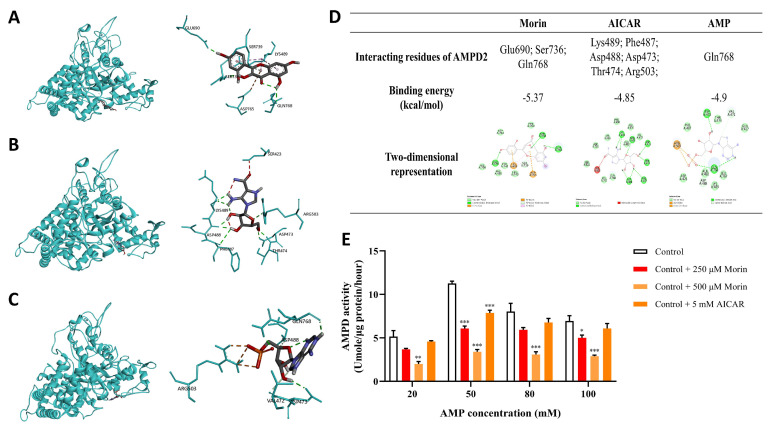
Morin inhibits AMPD activity, probably by interacting with AMPD2. (**A**–**D**) The molecular docking modes of morin, AICAR, and AMP in the binding site of AMPD2. (**E**) The enzyme activity of AMPD in vitro was measured by colorimetric methods (n = 4). * *p* < 0.05, ** *p* < 0.01, *** *p* < 0.001 compared with normal control group.

Molecular docking analysis was performed to evaluate the binding interactions of morin with AMPD2 (Figure 4A), along with PNC inhibitor AICAR (Figure 4B) and its natural substrate AMP (Figure 4C). The data showed that morin formed hydrogen bonds with the amino acid residues Glu690, Ser736, and Gln768 of AMPD2, which meant that morin might enter the substrate-binding domain to combine with AMPD2 specifically. Moreover, the binding free energy of AMPD2 with morin and AICAR was −5.37 and −4.85 kcal/mol, respectively, which were lower than or close to AMP (−4.9 kcal/mol), the typical substrate of AMPD2 (Figure 4D). The in vitro analysis further confirmed that 250 μM morin could have a significant inhibitory effect on AMPD activity with concentrations of AMP of 50, and 100 mM, while 500 μM morin had an inhibitory effect when AMP concentrations were at 20, 50, 80, and 100 mM. Moreover, 5 mM AICAR suppressed AMPD activity with an AMP concentration of 50 mM (Figure 4E).

### 2.3. Morin Improves Mitochondrial Energetic Disturbance Through AMPD2 Suppression in MPC5

**Figure 5:**
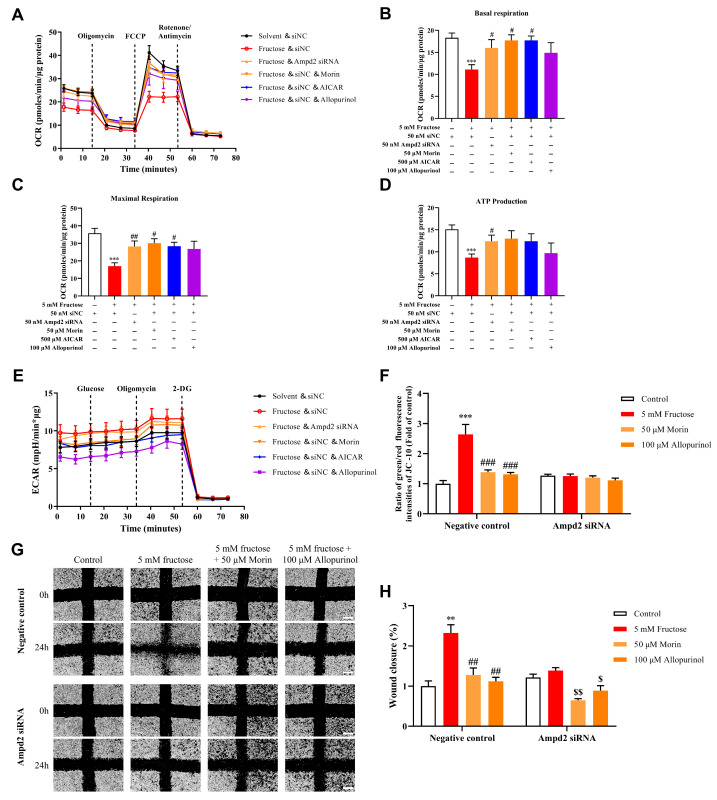
Effects of AMPD2 silencing on high-fructose-induced mitochondrial energetic disturbance and podocyte injury in MPC5. (**A**–**D**) OCR, basal respiration, maximal respiration, and ATP production were measured in podocytes transfected with *Ampd2* siRNA as well as negative control siRNA and then incubated with or without fructose, morin, AICAR, and allopurinol (n = 6–9). (**E**) ECAR was detected in podocytes transfected with *Ampd2* siRNA as well as negative control siRNA and then incubated with or without fructose, morin, AICAR, and allopurinol (n = 12). (**F**) Mitochondrial membrane potential was detected in podocytes transfected with *Ampd2* siRNA as well as negative control siRNA and then incubated with or without fructose, morin, and allopurinol (n = 7–8). (**G**,**H**) The migration ability was detected in podocytes transfected with *Ampd2* siRNA as well as negative control siRNA and then incubated with or without fructose, morin, and allopurinol. Scale bar = 500 μm (n = 3). The data conformed to normal distribution, and one-way ANOVA with Dunnett’s multiple comparison test was used to assess statistical differences. ** *p* < 0.01, *** *p* < 0.001 compared with normal control group, ^#^ *p* < 0.05, ^##^ *p* < 0.01, ^###^ *p* < 0.001 compared with fructose group (transfection with negative control siRNA), ^$^ *p* < 0.05, ^$$^ *p* < 0.01 compared with fructose group (transfection with *Ampd2* siRNA).

Next, we explored whether silencing AMPD2 could reverse the abnormalities in energy metabolism and podocyte injury induced by high fructose. The OCR (Figure 5A), basal respiration (Figure 5B), maximal respiration (Figure 5C), and ATP production (Figure 5D) of podocytes with fructose incubation were significantly lower than those of the control cells, which were significantly reversed in podocytes transfected with AMPD2 siRNA (Figure 5A–D). Meanwhile, morin and AICAR both significantly promoted OCR, as well as basal and maximal respiration rates in fructose-induced podocyte injury, whereas they did not exert significant promoting effects on ATP production (Figure 5A–D). ECAR analysis indicated that high fructose significantly induced glycolysis activation in podocytes (Figure 5E). Simultaneously, this abnormality was obviously improved by transfection with AMPD2 siRNA (Figure 5E). Morin, AICAR, and allopurinol could also reduce ECAR in podocytes induced by high fructose (Figure 5E). Accordingly, the promotion of mitochondrial membrane potential (MMP) and migration capacity was reduced by AMPD2 siRNA interference (Figure 5F–H). More meaningfully, the ameliorative effect of AMPD2 siRNA treatment on the changes in MMP and migration ability was shown to be further enhanced by morin (Figure 5F–H). These results preliminarily revealed that morin may function as an inhibitor to improve high-fructose-induced mitochondrial dysfunctions and abnormal energy metabolism by inhibiting AMPD2 activity.

### 2.4. Morin Ameliorates Podocyte Injury of High-Fructose-Fed Rats by Inhibiting AMPD Activity

**Figure 6:**
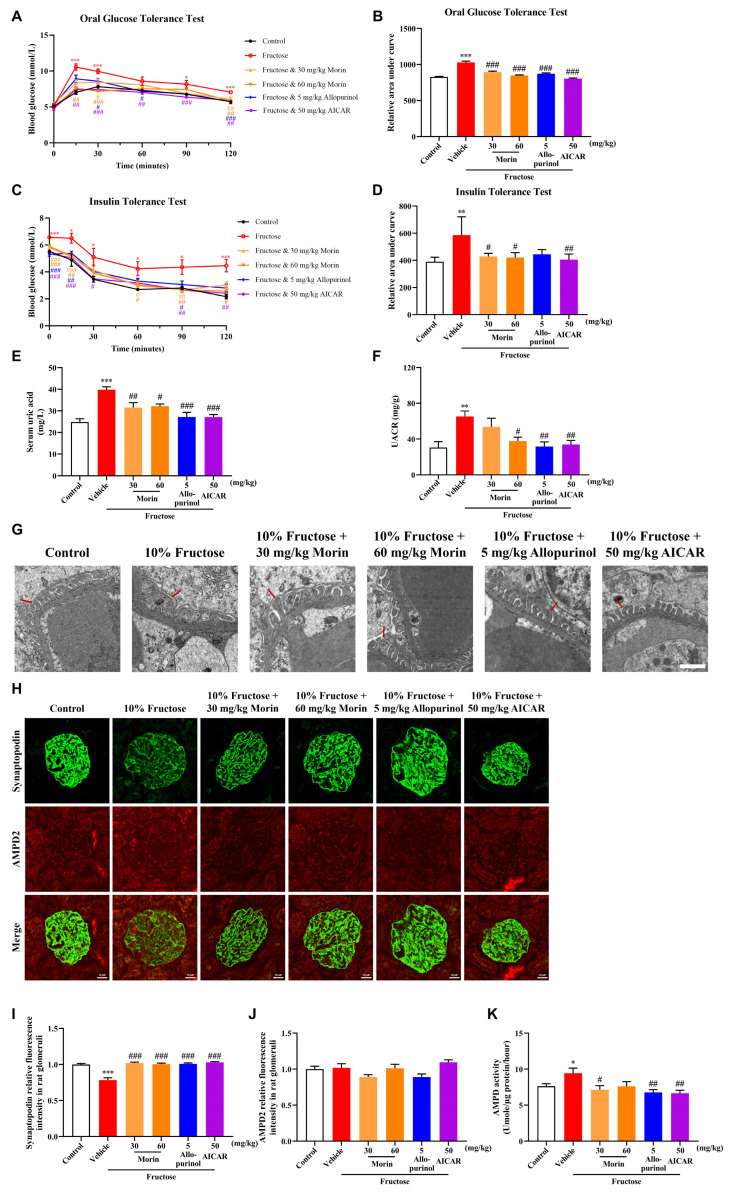
The effects of morin on the AMPD activity and podocyte injury in high-fructose-fed rats. (**A**,**B**) OGTT and (**C**,**D**) ITT of normal control group and high-fructose-induced metabolic syndrome rats fed with or without morin, allopurinol, and AICAR (n = 6). Serum uric acid (**E**) and UACR (**F**) were determined by biochemical kits (n = 5–6). (**G**) Transmission electron microscopy (TEM) of podocyte foot process effacement (arrow). Scale bar = 1 μm. (n = 3). (**H**–**J**) Representative immunofluorescence images of kidney glomeruli stained with synaptopodin (green) and AMPD2 (red). Scale bar = 20 μm (n = 6). (**K**) AMPD activity was detected in renal cortex using a phenol/hypochlorite reaction (n = 6). * *p* < 0.05, ** *p* < 0.01, *** *p* < 0.001 compared with normal control group, ^#^ *p* < 0.05, ^##^ *p* < 0.01, ^###^ *p* < 0.001 compared with fructose group.

Consistent with our previous studies [21], high fructose intake induced the development of metabolic syndrome, accompanied by glomerular podocyte injury in this study. Consistent with what we reported before [20], morin significantly improved glucose intolerance and insulin sensitivity in fructose-fed rats, as assessed by the oral glucose tolerance test (OGTT) and insulin tolerance test (ITT) (Figure 6A–D). Morin also restored the fructose-induced increase in serum uric acid levels and UACR (Figure 6E,F). Glomerular podocyte foot process effacement was observed in fructose-fed rats, which was significantly attenuated by morin (Figure 6G). Morin reversed the decrease in glomerular synaptopodin protein expression in rats with a high fructose diet (Figure 6H,I).

Moreover, no changes were observed in the expression of glomerular AMPD2 in rats with fructose-induced metabolic syndrome, with or without morin intervention, as shown by immunofluorescence assay (Figure 6H,J). Furthermore, the activity of AMPD in the renal cortex was increased by high fructose intake, which was significantly reversed by morin (Figure 6K). It was suggested that the inhibition of glomerular AMPD activity by morin was crucial for its ameliorative effect on podocyte injury in high-fructose-induced metabolic syndrome rats.

## 3. Discussion

Fructose consumption has increased dramatically over the past few decades and has been implicated in the high prevalence of metabolic syndrome [22] and subsequent glomerular dysfunctions [23,24]. Our previous study demonstrated that high fructose induces podocyte injury both in vivo and in vitro, the mechanism of which was related to oxidative stress, apoptosis, autophagy, and inflammation [25,26,27]. The present study unraveled a novel mechanism whereby fructose enhanced AMPD activity to dysregulate the mitochondrial energy metabolism, leading to podocyte injury. Morin effectively improved this mitochondrial disturbance and alleviated high-fructose-induced podocyte injury. Enzymatic assays and molecular docking analyses preliminarily suggested that morin might possibly inhibit AMPD activity through direct interaction with AMPD2 in the PNC. While previous studies on the PNC have largely been confined to skeletal muscle [7,28,29], and renal research has primarily focused on its role in ammoniagenesis [17,30], hepatic AMPD2 has been implicated in metabolic syndromes induced by monosodium glutamate or high-fat diets [13,31]. To our knowledge, this is the first study to elucidate the potential role of the PNC in high fructose-induced podocyte injury.

Morin, a multifunctional polyphenol, is reported to play a renal protective role in multiple animal models. Morin protects chicks from Aflatoxin B1-induced kidney injury by inhibiting inflammatory responses and oxidative stress [32], and mitigates cisplatin-induced nephrotoxicity through the inhibition of cytochrome P450 family 2 subfamily e polypeptide 1 (CYP2E1), NF-κB, P38 mitogen-activated protein kinase (MAPK), and apoptosis-related pathways [33]. While these studies reveal morin’s capacity to counteract oxidative stress and apoptosis, our investigation specifically focused on its potential to mitigate mitochondrial dysfunction. We found that morin attenuated high-fructose-induced mitochondrial function and alleviated podocyte injury. This protective mechanism aligns with the reports that polydatin and orientin preserve mitochondrial integrity in hyperglycemia-induced podocytes (MPC5) [34,35]. Collectively, these findings support the idea that morin attenuates podocyte injury, possibly by ameliorating mitochondrial dysfunction.

Energy homeostasis is mainly governed by mitochondria, which modulate multiple intracellular processes, including cell proliferation, calcium homeostasis, and oxidative stress [36]. In podocytes, which require high energy demands to maintain their specialized structure and function [37], ATP-dependent processes such as nephrin phosphorylation are essential for maintaining slit diaphragm integrity [38]. Our data demonstrate that ATP production was significantly decreased in high-fructose-treated podocytes, which was reversed by morin, further demonstrating the role of energy disturbance in podocyte injury. While differentiated mouse podocytes primarily rely on mitochondrial oxidative phosphorylation over glycolysis [39], undifferentiated podocytes contain fewer and less developed mitochondria and may depend more on glycolysis [40]. In this study, we found that glycolysis was significantly activated in high-fructose-induced podocyte injury, as indicated by increased ECAR. We propose that high fructose suppresses mitochondrial ATP generation while promoting glycolysis, potentially reflecting a dedifferentiation process. To sum up, these findings indicate that high fructose disturbs podocyte energy metabolism and that morin confers protection by restoring metabolic balance.

While PNC activation serves a protective role in maintaining the ATP energy balance in exercising skeletal muscles through AMPD-mediated AMP [41], we identified its pathological activation in high-fructose-induced podocyte injury. Despite similar ATP depletion, we observed partial PNC activation in podocytes, evidenced by selective AMPD upregulation, contrasting with the integrated pathway activation in muscle. It has been reported that AMPD2-generated ammonia may activate phosphofructokinase (PFK), the rate-limiting enzyme in glycolysis, in both vertebrate and invertebrate muscle and nervous tissue [11], while altered fumarate levels may disrupt TCA cycle flux. This metabolic reprogramming was reversed by AMPD2 silencing, evidenced by decreased OCR and increased ECAR in high fructose-treated podocytes, confirming the PNC’s role in renal pathogenesis. Enzymatic assays and molecular docking analyses provide preliminary evidence that morin may inhibit the activity of AMPD through direct interaction with AMPD2, the rate-limiting enzyme in the PNC. These collective observations raise the possibility that morin may alleviate podocyte energy disturbance through AMPD2 inhibition and subsequent modulation of PNC activity. In addition to its potential direct engagement with AMPD2, morin may regulate AMPD activity through complementary pathways. Previous studies have indicated that fructose-induced oxidative stress [42,43] can enhance AMPD activity [44,45]. Given morin’s documented antioxidant capacity [46], it may also suppress AMPD2 function through alleviating oxidative burden, suggesting a multifactorial mechanism underlying its protective effects.

The PNC may also influence energy metabolism through AMP-activated protein kinase (AMPK) signaling, a pivotal mediator of the cellular response to energy deficiency and mitochondrial insults [47]. Metformin improves insulin sensitivity and mitochondrial integrity in the skeletal muscle of fructose-fed rats by suppressing AMPD1, thereby increasing AMP levels and activating AMPK [48]. In addition, ADSL overexpression activates AMPK and ameliorates insulin resistance in high-fat-diet-induced obese mice [49]. Similarly, the AMPK agonist AICAR [50] improved high-fructose-induced energy disturbance in podocytes in our study. Therefore, this study provides potent evidence supporting the assumption that the PNC influences energy metabolism, partially by regulating AMPK. Although morin has been reported to activate AMPK by reducing oxidative stress in streptozotocin-treated pancreatic β-cells [51] and promoting autophagy in oxidized low-density lipoprotein-induced human umbilical vein endothelial cells [52], our data indicate it probably has a direct interaction with AMPD2 in fructose-treated podocytes. In light of the connection between the PNC and AMPK, further research is needed to determine whether AMPK acts downstream of the PNC in the modulation of podocyte mitochondrial energy metabolism.

Activated AMPD may contribute to intracellular uric acid accumulation, known to promote mitochondrial oxidative stress [53]. In this study, the unchanged expression and activity of ADSS in fructose-treated podocytes suggest that a potential uric acid conversion occurs due to IMP accumulation. This aligns with observations in hepatic model cells, where fructose increases intracellular uric acid levels and induces mitochondrial oxidative stress [54]. Similarly, our previous work demonstrated that nicotinamide adenine dinucleotide phosphate oxidase 4 mediated oxidative stress in high-fructose-injured podocytes, which was attenuated by allopurinol, a competitive inhibitor of xanthine oxidase [26]. Both morin, a known xanthine oxidase inhibitor [55], and allopurinol ameliorated mitochondrial dysfunction and podocyte injury in our models. This parallel suggests that AMPD2 activation may perturb purine metabolism alongside energy regulation. These findings suggest a potential role for the PNC in the regulation of podocyte energy homeostasis, with morin’s protective effects possibly involving AMPD2 inhibition and subsequent metabolic improvements.

This study elucidates the critical role of PNC-mediated metabolic reprogramming in high fructose-induced podocyte injury and proposes AMPD2 as a potential therapeutic target. However, several limitations should be considered. The research primarily focused on the AMPD2-mediated pathway without comprehensively evaluating other PNC components. Additionally, the experimental models employed, while capable of simulating metabolic stress, may not fully recapitulate the complex pathophysiology of human diseases. Furthermore, the mechanistic relationship between the PNC and energy-sensing pathways, such as AMPK, requires additional experimental validation. Future investigations should aim to delineate the molecular mechanisms underlying PNC-mediated regulation of podocyte differentiation, systematically evaluate the efficacy of morin and other PNC-targeting compounds across diverse pathological contexts, and explore the crosstalk between renal PNC function and metabolic homeostasis. These research directions will advance our understanding of how PNC dysregulation contributes to podocyte injury and glomerulopathy progression.

## 4. Materials and Methods

### 4.1. Reagents

For the animal experiments, fructose was purchased from Shandong Xiwang Sager Industry Co., Ltd. (Binzhou, China). Morin (C5297) was purchased from APExBIO (Houston, TX, USA). Allopurinol (A8003) was purchased from Sigma-Aldrich (Shanghai, China), and AICAR (Acadesine, SY005001) was purchased from ACCELA (Shanghai, China). A uric acid assay kit (C012-1-1), creatinine assay kit (C011-2-1), and rat microalbumin elisa kit (H127-1-2) were purchased from Nanjing Jiancheng Bioengineering Institute (Jiangsu, China). Electron microscope fixative (G1102-100ML) was obtained from Servicebio (Hubei, China). Hoechst 33258 (C1011) was purchased from Beyotime (Shanghai, China). Mouse anti-synaptopodin (sc-515842) was purchased from Santa Cruz Biotechnology (Santa Cruz, CA, USA) and rabbit anti-AMPD2 (PA5-29518) was purchased from Thermo Fisher Scientific (Waltham, MA, USA).

For cell experiments, fructose (F0127-100G), morin (480-16-0), and allopurinol (A8003) were all purchased from Sigma-Aldrich (Shanghai, China). AICAR (S1802) was purchased from Selleck (Housto, TX, USA). RPMI-1640 medium (L210KJ) was purchased from Shanghai BasalMedia Technologies Co., Ltd. (Shanghai, China). Fetal bovine serum (10099141C) was purchased from Gibco (Grand Island, NY, USA), and penicillin-streptomycin (S110JV) was purchased from Shanghai BasalMedia Technologies Co., Ltd. (Shanghai, China). Trypsin (25200056) was purchased from Gibco (Grand Island, NY, USA). Lipofectamine 2000 (11668019) was from Invitrogen (Carlsbad, CA, USA). Cell lysis buffer for Western and IP (P0013) was from Beyotime (Shanghai, China). Phenylmethanesulfonyl fluoride (PMSF) (P105539) was purchased from Aladdin (Shanghai, China). Pierce™ BCA protein assay kit (23225) was purchased from Thermo Fisher Scientific (Waltham, MA, USA). PVDF membranes (IPVH100010) were purchased from Millipore (Burlington, MA, USA). Mouse anti-AMPD1 (sc-393117) was purchased from Santa Cruz Biotechnology (Santa Cruz, CA, USA). Mouse anti-AMPD2 (67430-1-IG), rabbit anti-AMPD3 (23997-1-AP), and mouse anti-β-actin(60008-1-IG) were all purchased from Proteintech (Chicago, IL, USA). Rabbit anti-NPHS2 (ab50339) was purchased from Abcam (Cambridge, MA, USA). HRP-conjugated goat anti-mouse IgG(H+L) (SA00001-1) and HRP-conjugated goat anti-rabbit IgG(H+L) (SA00001-2) were purchased from Proteintech (Chicago, IL, USA). Trizol reagent (R401-01-AA), 5 × HiScript II Select qRT SuperMix (R222-01), and ChamQ SYBR^®^ qPCR Master Mix (Q311-02) were all purchased from Vazyme Biotech Co., Ltd. (Nanjing, China). A JC-10 kit (22204) was obtained from AAT Bioquest (Pleasanton, CA, USA).

### 4.2. Animals and Treatments

A total of 36 male Sprague Dawley rats (6 weeks old, 180–200 g) were purchased from Beijing Vital River Laboratory Animal Technology Co., Ltd. (Beijing, China). The protocol for animal experiments and treatment was checked and permitted by the Animal Ethical and Welfare Committee of Nanjing University (IACUC-2202011, 30 January 2022). Animals were kept in a constant-temperature environment with a 12 h light/dark cycle and fed with standard chow and water (ad libitum). After 7 days of acclimatization, rats were randomly divided into 2 groups: a control group (n = 6) with standard diet and drinking water, and a fructose group (n = 30) with standard chow and 10% fructose solution in drinking water (*w*/*v*). After 6 weeks, the fructose group was further divided into 5 subgroups (n = 6): fructose–vehicle, fructose with morin (30 and 60 mg/kg, intragastric administration), fructose with allopurinol (5 mg/kg, intragastric administration), and fructose with AICAR (50 mg/kg, intraperitoneal injection). Subsequent treatments were administered respectively to these subgroups for the next 4 weeks. All dosages were based on previous studies [20,21,25,56,57,58,59]. Our previous results showed that allopurinol effectively alleviated oxidative stress and podocyte apoptosis in fructose-fed rats [25,26]. Nissim et al. [60] demonstrated that 5-Aminoimidazole-4-carboxamide-1-β-D-ribofuranoside (AICAR) inhibited the PNC in renal tubules from chronically acidotic rats and reduced ammonia production, suggesting there was a potential clinical use for AICAR in renal failure. Therefore, we selected allopurinol and AICAR as positive drugs in this study. Morin and allopurinol were mixed by ultrasonic waves, suspended, or dissolved in normal saline. AICAR was dissolved in PBS by ultrasonic vibration and then filtered to remove bacteria. At week 11, each rat was placed in a metabolic cage to collect 24 h urine before being sacrificed. Urine samples were centrifuged at 3000 *g* for 10 min at 4 °C and stored at −80 °C. After rats were anesthetized with sodium pentobarbital (50 mg/kg, intragastric administration), blood samples were collected, centrifuged to extract serum, and then kept at −80 °C for later analysis as we reported previously [21]. Meanwhile, the kidney tissues were isolated on ice rapidly and divided into several portions, which were preserved in paraformaldehyde, electron microscopy fixative, or directly frozen in liquid nitrogen and then stored at −80 °C for different subsequent analyses. The isolation of rat glomeruli was carried out as follows: the renal cortex was cut into small pieces and then cooled. Hank’s Balanced Salt Solution was used to grind the pieces on a 180 μm stainless mesh sieve under pressure. The tissue was subsequently filtered through 180 μm and 150 μm stainless mesh sieves, each for three repetitions. After centrifugation at 4 °C, 2500 rpm for 10 min, the sediment collected in the solution was considered to be glomeruli and was stored at −80 °C.

### 4.3. OGTT and ITT

OGTT and ITT were performed in line with our previous studies [61]. In brief, after fasting for 14 or 6 h, rats were administered glucose orally (1.5 g/kg body weight) or injected intraperitoneally with recombinant human regular insulin (0.8 U/kg body weight), respectively. The glucose levels of rat tail vein blood were measured at 0, 15, 30, 60, 90, and 120 min by glucometer (One Touch^®^ Ultra, Roche Diagnostics GmbH, Mannheim, Germany).

### 4.4. Biochemical Analysis

After rats were anesthetized with sodium pentobarbital, blood samples were collected from the rats’ abdominal aorta. After allowing the collected blood samples to stand at room temperature for 30 min, they were then centrifuged at 4 °C, 3000 rpm for 10 min to obtain the serum. The levels of serum uric acid, urine creatinine and urine microalbumin were detected by commercially available biochemical kits following the manufacturer’s instructions.

### 4.5. Transmission Electron Microscopy Analysis

Transmission electron microscopy analysis was performed as described previously [25]. Briefly, renal cortex cubes of 1–2 mm were cut from fresh rat kidneys and immediately placed in electron microscope fixative for 2 h at room temperature and away from light, then the samples were transferred to 4 °C for preservation. Glomerular ultrathin sections were examined with a JEOL-JEM 1010 transmission electron microscope (Jeol, Tokyo, Japan).

### 4.6. Immunohistofluorescence Assay

The method of Immunohistofluorescence analysis was performed as in previous studies [62] to detect the expression of synaptopodin and AMPD2 in rat glomeruli. Briefly, rat renal cortex tissues were fixed with 4% paraformaldehyde, embedded in paraffin, and then sectioned transversely. Sections were incubated overnight with primary antibodies at 4 °C after deparaffinizing, antigen retrieval, and blocking, then the sections were washed 3 times with PBS and incubated with secondary antibodies for 2 h at room temperature away from light. The nucleus was stained with Hoechst (1:2500). Finally, the stained renal cortex sections were examined by a confocal laser scanning microscope (Lei TCS SP8-MaiTai MP; Leica, Wetzlar, Germany).

### 4.7. Cell Culture and Treatment

The conditionally immortalized MPC5 was purchased from Fuheng Biology Company (Shanghai, China) and cultured in RPMI-1640 medium supplemented with 10% fetal bovine serum and 1% penicillin-streptomycin at 37 °C. Upon reaching 80–90% confluency in the culture flask, cells were digested with trypsin and then plated in plastic culture plates for various experiments. Podocytes were treated with or without 5 mM fructose in the presence or absence of 25, 50 μM morin, 100 μM allopurinol, or 500 μM AICAR for 72 h to measure protein levels, mRNA levels, and mitochondrial membrane potential, etc. Drug concentration selection for the treatment of podocytes was based on previous reports [25,59,63,64,65,66].

*Ampd2* siRNA and negative control siRNA were purchased from Biotend company (Shanghai, China). The primer sequences are shown in Table 1. Before transfection, podocytes were seeded in 6-well plastic culture plates and allowed to reach 50–60% confluency. *Ampd2* siRNA (50 nM) and negative control siRNA (50 nM) were transfected into podocytes with Lipofectamine 2000 (Invitrogen, Carlsbad, CA, USA). After 6 h of transfection, podocytes were cultured in RPMI-1640 medium for 48 h, and the transfection efficiency was assessed by Western blot analysis.

### 4.8. Wound-Healing Assay

Podocytes (1 × 10^5^ cells/well) were cultured in 6-well plates and treated as described above. When podocytes had grown to confluence, a 200 μL pipette tip was used to make a scratch. After wounding, the detached cells were washed away with PBS, and the cells were allowed to migrate in serum-free 1640 medium for 24 h. Photos were taken with phase-contrast microscopy (Olympus, Tokyo, Japan) under a × 4 objective at 0 and 24 h. The wound closure area was measured with Image J (version 1.53t, National Institutes of Health, Bethesda, MD, USA). The sample size for the wound-healing assay is n = 3.

### 4.9. Western Blot Assay

The total protein extracted from the podocytes and cultured in 6-well plates was prepared using cell lysis buffer for Western and IP, supplemented with 1 mM PMSF. The protein concentration was determined using the Pierce™ BCA protein assay kit according to the manufacturer’s instructions. After adjusting the protein concentration to the same level using cell lysis buffer, 5× loading buffer, and mercaptoethanol were added. The samples were then thoroughly mixed and kept in boiling water for 10 min. Equal amounts of proteins were separated on 7.5% or 10% SDS-PAGE and then transferred onto PVDF membranes. The membranes were blocked with 5% fat-free milk (*w*/*v*) for 1 h at room temperature and then incubated with primary antibodies overnight at 4 °C. The dilution ratios of all primary antibodies used in this experiment were as follows: anti-AMPD1 (1:1000), anti-AMPD2 (1:1000), anti-AMPD3 (1:1000), anti-NPHS2 (1:1000), and anti-β-actin (1:5000). After washing, the membrane was incubated with the corresponding secondary antibody (1:5000) according to the species of primary antibody for 1 h. The bands were visualized using an enhanced chemiluminescence system (Tanon Science & Technology Co., Ltd., Shanghai, China) and analyzed by Image J. The sample sizes for the Western blot assay were n = 8 for measuring podocin expression and n = 6 for measuring other proteins.

### 4.10. Quantitative Reverse Transcription Polymerase Chain Reaction (qRT-PCR) Assay

Total RNA was extracted from podocytes cultured in 6-well plates using Trizol reagent. Reverse transcription reactions of total RNA to cDNA were conducted using 5 × HiScript II Select qRT SuperMix. The reaction program was set as 50 °C for 15 min, followed by 85 °C for 2 min. The resulting cDNA was stored at −20 °C. The qRT-PCR reactions were carried out with ChamQ SYBR^®^ qPCR Master Mix in a CFX96 Real-Time PCR Detection System (Bio-Rad, Hercules, CA, USA) with the reaction program set as 95 °C for 3 min, followed by 40 cycles of a two-step amplification program (95 °C for 10 s and 60 °C for 30 s). All the above steps were carried out according to the manufacturer’s instructions. Primer sequences were also shown in Table 1. To acquire quantitative results, the Ct (2^−ΔΔCt^) method [67] was employed to measure the relative expression levels of targeted genes examined in this research, after normalizing their expression levels to the expression level of β-actin. The sample sizes for qRT-PCR are n = 3.

### 4.11. JC-10 Assay

The mitochondrial membrane potential of podocytes was measured with the JC-10 kit. In brief, cells were treated with test compounds as described above, and then the JC-10 working solution was added to the cell plate. After a period of incubation, the fluorescence change was monitored at Ex/Em = 490/525 nm (FITC channel) and 540/590 nm (TRITC channel) using a fluorescence microplate reader (CLARIO star, BMG Labtech, Ortenberg, Germany) for ratio analysis. The sample size for the JC-10 assay was n = 5–6.

### 4.12. Seahorse Measurement

Podocytes were cultured to the appropriate confluency and then seeded in XFe96 cell culture microplates (101085-004, Seahorse Bioscience, Agilent Technologies, Santa Clara, CA, USA) as described above at a density of 20,000/well. After overnight hydration of the sensor cartridge, adding compounds to the reagent ports, and changing the medium to assay medium, energy metabolism analysis was performed with the XFe96 Extracellular Flux Analyzer (Seahorse Bioscience, Agilent Technologies). Pierce™ BCA protein assay kit (Thermo Fisher Scientific, Waltham, MA, USA) was used for data normalization.

### 4.13. Determination of AMPD Activity

AMPD activity in podocytes was determined as described, with some modifications [68,69]. Both cells and tissues were lysed with Buffer A (1 mM PMSF, 200 mM sucrose, 150 mM KCl, 20 mM imidazole in 50 mM dibasic sodium phosphate-citric acid buffer, pH = 7), which was followed by sonication. The mixture was centrifuged at 12,000 rpm for 10 min at 4 °C to obtain the supernatant for the measurement of enzyme activity. The protein concentration in the supernatant was measured with the Pierce™ BCA protein assay kit. Briefly, 50 μL of supernatant from each sample was added to a 96-well plate, then 100 μL of the reaction system (50 mM KCl, 100 mM AMP in 50 mM dibasic sodium phosphate-citric acid buffer, pH = 7) was added to each well. For each protein extract, a blank well without AMP was included for background quantification. After incubation at 37 °C for 30 min, 100 μL of reagent A (100 mM phenol and 200 mM sodium nitroprusside in H_2_O) was added to each well. In total, 50 μL of the previous reaction was transferred to another well of the 96-well plate containing 100 μL of Reagent B (125 mM sodium hydroxide, 200 mM dibasic sodium phosphate, 0.1% sodium hypochlorite in H_2_O). After 1 h of incubation at 25 °C, the absorbance was measured at 625 nm by using a microplate reader (CLARIO star, BMG Labtech, Ortenberg, Germany). Ammonium sulfate was used to construct ammonia standard curves to quantify the ammonia production from each well. The sample size for the determination of AMPD activity was n = 5–6.

### 4.14. Determination of ADSS and ADSL Activity

The activity of ADSS and ADSL were measured according to the methods used in previous studies [70,71]. Podocytes were treated with test compounds for 72 h. Before harvesting, cells were washed twice with PBS and lysed with 20 mM potassium phosphate buffer (containing 1 mM DTT, pH = 7.0) on ice for 10 min, then transferred to −20 °C and frozen for 5 min. Samples were centrifuged at 12,000 rpm at 4 °C for 10 min to obtain supernatants, and then the protein concentration was measured. For ADSS, the reaction system contained 50 μL supernatant and 150 μL of 20 mM potassium phosphate buffer (pH = 7.5, containing 10 mM aspartic acid, 0.3 mM IMP, 0.12 mM GTP, and 2 mM MgCl_2_). For ADSL, the reaction system contained 50 μL supernatant and 150 μL of 20 mM potassium phosphate buffer (pH = 7.0, 0.1 mM adenylosuccinate). Reactions were incubated at 37 °C for 30 min, and the absorbance at 280 nm was measured by using a microplate reader at 0 and 30 min. The sample sizes for the determination of ADSS and ADSL activity were n = 4–6.

### 4.15. Inhibitory Effect of AMPD Activity by Morin

Normal cells were lysed with Buffer A to obtain total protein extraction. The measurement of protein concentration in the supernatant was performed as described in 4.13. For each reaction group, the 200 μL system contained 36 μL supernatant, 10 μL 1 mol/L KCl, 40 μL AMP (0, 20, 50, 80, 100 mM), and 114 μL 50 mM dibasic sodium phosphate-citric acid buffer (pH = 7), in the absence or presence of 250 μM morin, 500 μM morin, and 5 mM AICAR. Reactions were conducted at 37 °C for 30 min, and the absorbance at 625 nm was measured.

### 4.16. Data Mining in HPA Database

The mRNA and protein expression levels of AMPD1, AMPD2, and AMPD3 in human tissue samples were obtained from the HPA database (The Human Protein Atlas, https://www.proteinatlas.org/) [72,73] and accessed on 27 May 2024. The data showed that AMPD1 was barely expressed in the kidney.

### 4.17. Molecular Docking

Because the 3D structure of human AMPD2 has not been analyzed, we chose Modeller 9v22 software [74,75,76,77] to construct the 3D structure of human AMPD2 by using the X-ray crystal structure of Arabidopsis-derived AMPD2 (PDB ID 2A3L) [78] as a template, and the sequence similarity between the template and human AMPD2 was 54%. The 3D structure of morin, AICAR, and AMP were obtained from the PubChem database (https://pubchem.ncbi.nlm.nih.gov/) [79] accessed on 29 January 2022. Before docking, the polar hydrogen and Gasteiger charges were added to receptors and small molecules using AutoDockTools1.5.6, and molecular docking was conducted by AutoDock4.2.6 (Center for Computational Structural Biology, ccsb, California, CA, USA) [80]. The dimensions of the grid box for AMPD2 were set to 40 Å, 40 Å, and 40 Å, and the grid spacing value was set to 0.375 Å. Lamarckian Genetic Algorithm with 250 runs was applied with 25,000,000 evaluations each. The interacting amino acids were identified using AutoDockTools. The visualization and imaging were created by Discovery Studio Visualizer Client 2020.

### 4.18. Statistical Analysis

All data were analyzed by using GraphPad Prism 8.0 software (San Diego, CA, USA) and shown as mean ± SEM. Prior to statistical analysis, data normality was confirmed using the Shapiro–Wilk test. For normally distributed data, the unpaired t-test or one-way ANOVA with Dunnett’s multiple comparison test was used to assess differences between two groups or among multiple groups. For non-normally distributed data, the Mann–Whitney test or Kruskal–Wallis test with Dunnett’s multiple comparison test were applied. *p* < 0.05 was considered statistically significant.

## 5. Conclusions

In conclusion, our findings indicated that morin effectively attenuated the high fructose-induced disturbance in mitochondrial energy metabolism by inhibiting AMPD2 activity in the PNC, as validated both in vivo and in vitro. Notably, the present study indicated that the PNC is likely to be an upstream mediator of mitochondrial energy metabolism to modulate podocyte structure and function. We further identified morin and AICAR as potential inhibitors of AMPD2, suggesting AMPD2 as a promising therapeutic target for podocyte injury associated with long-term fructose intake. This study provides novel insights into the renal protective role of morin in mitigating podocyte injury under high fructose-induced metabolic stress.

## Figures and Tables

**Table 1:** The primer sequences and siRNA sequences used in this study.

Gene	Forward (5′-3′)	Reverse (5′-3′)
*Ampd2*	TTGATAGCGTGGATGATGAG	CCCGTGGGAGATGTTCTCGG
*Ampd3*	GTTGGCGGAGAAGGTGTTTG	CTGCGACCGGATCATCTTGAA
*Adss*	TGCAAACGCAGCATTGTTAGA	GGAAAGGCACCAATACCAACTC
*Adsl*	TACTTCAGCCCCATCCACTC	TCACTGTAACCGGGTTCTCC
*β-actin*	GGGAAATCGTGCGTGAC	AGGCTGGAAAAGAGCCT
*Ampd2* siRNA	GUGCAUGCGGACAGGAAUATT	UAUUCCUGUCCGCAUGCACTT
*Ampd3* siRNA	GGAAGAUGCUGGAGAACAUTT	AUGUUCUCCAGCAUCUUCCTT
Negative control siRNA	UUCUCCGAACGUGUCACGUTT	ACGUGACACGUUCGGAGAATT

## Data Availability

The original data presented in the study are openly available in FigShare at https://doi.org/10.6084/m9.figshare.30571082.

## Abbreviations

The following abbreviations are used in this manuscript:

UACR	urinary albumin to creatinine ratio
AMPD	adenosine 5′-monophosphate deaminase
MPC5	mouse podocyte clone-5
PNC	purine nucleotide cycle
OCR	oxygen consumption rate
2-DG	2-deoxyglucose
ADSS	adenylosuccinate synthetase
ADSL	adenylosuccinate lyase
ECAR	extracellular acidification rate
AICAR	5-Aminoimidazole-4-carboxamide-1-β-D-ribofuranoside
OGTT	oral glucose tolerance test
ITT	insulin tolerance test
CYP2E1	cytochrome P450 family 2 subfamily e polypeptide 1
MAPK	mitogen-activated protein kinase
PMSF	phenylmethanesulfonyl fluoride
qRT-PCR	quantitative reverse transcription polymerase chain reaction
